# Inverse comorbidity: the power of paradox in the advancement of science

**DOI:** 10.15256/joc.2013.3.19

**Published:** 2013-03-22

**Authors:** Rafael Tabarés-Seisdedos, Jose M. Valderas

**Affiliations:** ^1^Department of Medicine, University of Valencia, CIBERSAM, Valencia, Spain; ^2^LSE Health, Department of Social Policy, London School of Economics and Political Science, London, UK; ^3^Department of Primary Care Health Sciences, University of Oxford, Oxford, UK

Research on comorbidity and multimorbidity is finally receiving the attention it deserves, particularly considering the magnitude and impact they have on health and the delivery of healthcare [1, 2]. Numerous studies have demonstrated that individuals with Down’s syndrome, Parkinson’s disease, schizophrenia, diabetes, anorexia nervosa, Alzheimer’s disease, allergy related diseases, multiple sclerosis or Huntington’s disease (among other health problems) are protected against many forms of cancer, including solid tumors, smoking-related tumors and prostate cancer. This apparent anti-cancer effect, which we have termed *inverse cancer comorbidity*, has been observed in many serious CNS and immune disorders, and is the subject of active research [3–5].

## Paradoxical nature of inverse comorbidity

While paradoxes are concepts that violate common sense, they have been an important source of inspiration and scientific progress in logic, mathematics, physics and economics. For example, the Viennese mathematician Kurt Gödel’s (1906–1978) *Theorem of Incompleteness in Arithmetic* stated that all mathematical systems have propositions that are impossible to prove or reject, thus affirming the limited and incomplete nature of mathematical knowledge. Gödel’s incompleteness theorem has influenced fields beyond mathematics and logic, contributing to the development of computational machines and theory of mind, among others [6]. Counterintuitive realities also have a long tradition in biology, medicine and other life sciences. An example of one such peculiarity in immunology is the presence of “elite controllers” –rare individuals infected with human immunodeficiency virus (HIV) whose immune systems are capable of spontaneously controlling HIV-1 replication for decades without the assistance of antiretroviral medication. Indeed, for a long time the existence of these elite controllers was considered proof that acquired immunodeficiency syndrome (AIDS) was not viral. In recent years, various genome-wide association studies (GWAS) have demonstrated that polymorphism within the human leukocyte antigen (HLA) class I locus is the primary host genetic determinant of the rate of progression of HIV infection to AIDS – which explains the dramatic differences observed in untreated disease outcome [7, 8]. A deeper understanding of innate immunity and early immune responses to HIV-1 could lead to the development of an effective HIV-1 vaccine [9, 10].

Another example of a medical paradox relates to supercentenarians. These people, who live to the age of at least 110, are exceedingly rare: a mere seven of every 1,000 people reach the century milestone in industrialized countries [11, 12], and only 70 supercentenarians (of whom 65 are women) have been validated worldwide by the Los Angeles Gerontology Research Group [13]. Given that genetic contribution is more pronounced at an older age, exceptional longevity and healthspan may be attributable to disease-associated and protective variants in the genome [14]. Longevity genes encode proteins involved in biological processes, including lipid-protein metabolism (e.g. apolipoprotein E); signaling of growth hormone 1/insulin-like growth factor 1/insulin; DNA damage, signaling, and repair; and pro/antioxidant pathways [15]. These molecular mechanisms also have a pivotal function in cancer and neurodegeneration. In this way, supercentenarians may be the key to a better understanding not only of the biological and non-biological determinants of longevity but also of cancer and CNS disorders; knowledge that could be used to improve the quality of life of people in their latter years.

The traditional understanding of diseases in dichotomous (present versus absent), and hence, opposing terms, has led medical practice to focus on specific diagnostic categories or groups of disorders with overlapping phenotypes or pathophenotypes (e.g. neurodegenerative disorders with the same cognitive impairments). Although this reductionist view is practical at a clinical level, it ignores the fact that the boundaries between diseases are blurred in terms of genetics or proteomics [16]. By contrast, the more contemporary, cross-sectional perspective of disease–disease associations (positive or direct comorbidities) and counter-associations (inverse comorbidities) is based on the premise that the causes and pathogenic mechanisms of medical conditions are common to multiple categories. This viewpoint has led to the development of a highly valuable network of disorders (phenotypic comorbidity network) [17, 18]. Moreover, several systems studies have discovered novel disease associations based on either shared disease-causing genes or overlapping pathways [19]. For example, genetic susceptibility to seven autoimmune and inflammatory (immune-mediated) diseases (celiac disease, Crohn’s disease, multiple sclerosis, psoriasis, rheumatoid arthritis, systemic lupus erythematosus, and type 1 diabetes) accounts for 47 of 107 (44%) immune-mediated–disease risk single-nucleotide polymorphisms (SNPs) [20, 21]. This finding might explain why these disorders co-exist in certain populations. Evidence from studies examining association, gene expression and endophenotype have identified at least three risk genes located on chromosome 8p *NRG1, DPYSL2, PPP3CC*) affecting the expression of specific phenotypes – including those of autism, schizophrenia, Alzheimer’s disease, Parkinson’s disease, prostate cancer and several types of epithelial cancers (breast, pancreatic and colon) – across the nosological boundaries [3–5]. Therefore, network methods that integrate different genetic, proteomic and metabolic datasets related to comorbidities (or spanning more than one disease) are likely to not only improve our understanding of disease etiology and of phenotype-biomarker-genotype associations [16] but also help build a bridge between medical disciplines by promoting scientific integration.

We believe that inverse comorbidity represents an unprecedented opportunity to gain insight into the pathogenesis of many serious diseases, and that understanding why certain individuals diagnosed with specific disorders are protected against other medical conditions could help to develop new and improved treatments [5, 22].
